# Relationships between the *Advanced Nursing Process* quality and nurses' and patient' characteristics: A cross‐sectional study

**DOI:** 10.1002/nop2.405

**Published:** 2019-11-08

**Authors:** Claudia Leoni‐Scheiber, Hanna Mayer, Maria Müller‐Staub

**Affiliations:** ^1^ Institute of Nursing Science University of Vienna Vienna Austria; ^2^ Lectoraat Nursing Diagnostics HANZE University Groningen Groningen Netherlands

**Keywords:** advanced nursing process, correlation, cross‐sectional study, instrument quality of nursing diagnoses, interventions and outcomes (q‐dio), nurse attitudes, nursing knowledge, nursing records, organizational factors, positions on nursing diagnoses scale (pnd), record review

## Abstract

**Aim:**

This study aimed to assess (a) nurses' knowledge and their attitude towards the Advanced Nursing Process—nursing assessment, diagnoses, interventions, outcomes, (b) the quality of the Advanced Nursing Process and (c) relationships with patient characteristics.

**Design:**

A cross‐sectional, descriptive correlational study was performed.

**Methods:**

Ninety‐two registered nurses and ninety nursing records of six hospital wards were included. In January 2016, a knowledge test, a self‐assessment tool for measuring nurses' attitude (PND) and the *Quality of Diagnoses, Interventions and Outcomes Revised* instrument (Q‐DIO R) were applied. The correlations between nurses' knowledge, attitude, patient characteristics, organizational factors and the Advanced Nursing Process quality were investigated.

**Results:**

Nurses demonstrated low levels of knowledge, positive attitudes and an average Advanced Nursing Process quality. Accurate nursing diagnoses were strong and highly significantly related to effective nursing interventions and better nursing‐sensitive patient outcomes. A higher proportion of registered nurses was related to better nursing outcomes.

## 1 INTRODUCTION

Patients benefit from the Advanced Nursing Process: By its use, nursing interventions were more effective, better nursing outcomes were reached, patients felt more comfortable and patient safety increased (Müller‐Staub, Needham, Odenbreit, Lavin, & Achterberg, 2008; Pèrez Rivas et al., 2016). However, its implementation in practice fails often (Pereira et al., 2015) because of nurses' poor knowledge regarding diagnostic concepts (Patiraki, Katsaragakis, Dreliozi, & Prezerakos, 2017) and on evidence‐based nursing interventions and outcomes. Nurses' attitude towards the Advanced Nursing Process is often ambivalent or negative (Romero‐Sànchez et al., 2013) and organizational factors like bed occupancy rate, skill‐ and grade‐mix and length of stay (LOS) are hindering its implementation (Conrad, Hanson, Hasenau, & Stocker‐Schneider, 2012). This is supplemented by increasing numbers of elderly, multimorbid patients with complex care needs and a simultaneous trend towards hiring fewer registered nurses (RNs) and higher staff turnovers (Buchan, Shaffer, & Catton, 2018).

### 1.1 Background

The Advanced Nursing Process is a specific form of the “traditional nursing process.” It consists of valid assessment tools and on defined, valid concepts of nursing diagnoses (NDs), nursing interventions and nursing outcomes “that are rooted in scientifically based nursing classifications” (Müller‐Staub, Abt, Brenner, & Hofer, 2015, p. 13). The NNN taxonomy (Johnson et al., 2011), consisting of the nursing diagnoses classification of NANDA International, the Nursing Outcomes Classification (NOC) and the Nursing Interventions Classification (NIC), meets most of the validity and reliability criteria for classifications systems (Müller‐Staub, Schalek, & König, 2017). The quality of the Advanced Nursing Process is not only determined by its documentation in nursing records, but rather by its internal coherence. Coherence means that accurately formulated NDs are correctly linked with effective nursing interventions and matching outcomes. The accuracy of NDs relies on the PES format (problem definition, aetiological factors and signs/symptoms) and is the initial point for choosing effective nursing interventions and outcomes showing the effectiveness of interventions. The quality of NDs also depends on their relevance, frequency and variety to describe patients' care needs correctly. Fewer NDs and a smaller variety could depend on the patient and the reason for hospital admission or/and could also indicate lower diagnostic quality (Johnson et al., 2011; Müller‐Staub et al., 2009).

The quality of the Advanced Nursing Process is influenced by different factors: firstly, by nurses' knowledge and attitude. For the implementation of the Advanced Nursing Process, nurses need patient‐related knowledge about the person and situation, clinical decision‐making competence including diagnostic reasoning and knowledge about validated concepts of the NNN taxonomy (Lunney, 2007; Paans, Nieweg, Schans, & Sermeus, 2011). The professionals' behaviour (the implementation of the Advanced Nursing Process) is influenced by their individual intention to act. This intention, in turn, is mainly determined by nurses' attitude referred to their behaviour (Ajzen, 2012). Secondly, the Advanced Nursing Process is influenced by patient characteristics such as age, admission to medical speciality and LOS. Further, also organizational factors could influence the Advanced Nursing Process quality. Several studies addressed the relationships between nurses' knowledge and attitude towards the Advanced Nursing Process (Kim & Shin, 2016; Paans et al., 2011), respectively, nurses' knowledge or attitude and organizational factors (Lumillo‐Gutierrez et al., 2018; Okaisu, Kalikwani, Wanyana, & Coetzee, 2014) and connections between knowledge, attitude and organizational factors (Kebede, Endris, & Zegeye, 2017). Studies focusing on all these relationships with the entire Advanced Nursing Process—nursing assessment, diagnoses, interventions and outcomes—are missing.

### 1.2 Aims and research questions

The overall objective was to measure the effects of an educational intervention on nurses' knowledge, attitude and the Advanced Nursing Process quality by performing an experimental intervention study (Leoni‐Scheiber, Mayer, & Müller‐Staub, 2019). The aim of this paper is to report baseline findings and relationships between nurses' knowledge and attitude towards the Advanced Nursing Process; the quality of NDs, interventions and outcomes; and patient characteristics (gender, age, LOS).

Research questions:

1. How is nurses' knowledge and attitude towards the implementation of the Advanced Nursing Process?
2. What is the quality of the Advanced Nursing Process as measured by (a) the accuracy of NDs, (b) effectiveness of nursing interventions, (c) the quality of nursing outcomes and (d) frequency and variety of NDs?
3. Do correlations exist between nurses' knowledge and attitude with the quality of the Advanced Nursing Process?
4. Do correlations exist between patient characteristics and the quality of the Advanced Nursing Process?

## 2 DESIGN

A cross‐sectional, descriptive correlational design was carried out to investigate the relations between nurses' and patient characteristics and the quality of NDs, interventions and outcomes. Therefore, a knowledge test for nurses, a self‐assessment instrument measuring nurses' attitude towards the Advanced Nursing Process and a tool for demographic and organizational factors were applied. The Advanced Nursing Process quality was measured by using the Q‐DIO R instrument. The study was conducted in a Swiss 260 bed hospital in three departments (medical, surgical, acute geriatric).

## 3 METHODS

### 3.1 Sample/Participants

Nurses and nursing records of six hospital wards were included. The inclusion criterion was general wards (two medical, two surgical and two acute geriatric). In January 2016, using stratified convenience sampling, a third of the RNs from these wards (*N* = 34) was selected and asked to fill out the knowledge test. Eligibility criteria for nurses were as follows: holding a position as ward manager; nurse instructor; Advanced Nursing Process mentor; or being a regular RN applying the Advanced Nursing Process covering all shifts and speaking German fluently. The composition of this group based on her specific responsibility for the Advanced Nursing Process. Later, this group would participate in an educational intervention study. All RNs of the six wards (*N* = 99), not only the group described above, were included for measuring their attitude towards the Advanced Nursing Process. The reason for including all nurses in the attitude measurement was that all RNs would later participate in case meetings on their wards for a following study.

The quality of the Advanced Nursing Process was evaluated by nursing record audits of the six wards in January 2016 (15 per ward, in total 90 records). The nursing process documentation was part of the Electronic Health Record (EHR) that included 43 NANDA‐I NDs with related nursing outcomes and interventions formulated according to Doenges, Moorhouse, and Murr (2014). A decision support system was not available in the EHR at that time. The nursing records of each patient were created by the responsible RN at any shift. Therefore, it is a cumulative product created by several nurses. Besides the nursing assessment and care plan, other nursing record content (e. g. nursing notes/report, performance descriptions)—of the duration of four days after stating the Nursing Diagnosis—was included. Record inclusion criteria were as follows: containing an individual care plan with at least one ND and patients' LOS for at least 4 days. A random sample of records was drawn by random generator (https://www.random.org/).

### 3.2 Data collection

#### 3.2.1 Measurement instruments

A knowledge test was developed and pilot‐tested in a previous educational training. After minor adjustments, it contained seven items: Six qualitative knowledge questions (e.g. definition of nursing diagnosis, meaning and function of the PES format, coherence between NDs, interventions and outcomes) and one self‐evaluation item on nurses' knowledge. Results of this self‐evaluation item were compared with the total score of the knowledge test (maximum 58 points).

Nurses' attitude towards NDs was measured by the *Positions on the Nursing Diagnoses (PND)* scale (Lunney & Krenz, 1994). It contains 20 contrasting pairs of attitude adjectives on a seven‐point Likert scale (e.g. easy—difficult, important—unimportant). Its total score ranges from 20–140; the more positive the attitude, the higher the total score. The German PND (Lunney, 2007) was previously evaluated in a quasi‐experimental study (Leoni‐Scheiber, Gothe, & Müller‐Staub, 2016). The original PND was repeatedly tested and showed good results, for example content validity 90 per cent agreement (Lunney & Krenz, 1994), test–retest reliability 0.90 [95% CI (0.87–0.92)], internal consistency by Cronbach's alpha 0.96 (Romero‐Sánchez et al., 2013), construct validity by confirmatory factor analysis (≥0.96) (D’Agostino et al., 2016).

To assess the quality of the Advanced Nursing Process, the Quality of Diagnoses, Interventions and Outcomes (Q‐DIO) instrument (Müller‐Staub et al., 2009, 2010) was revised and applied for the record audit. The Q‐DIO R was expanded from 29–35 items, for example the item “congruence between assessment and nursing history data and the complexity of the case” has been added. The scales remained, a three‐point scale (0–2), was used for the twelve nursing assessment items, a five‐point scale (0–4) for the other three sub‐concepts (NDs as product, nursing interventions and nursing outcomes). The higher the total score of each sub‐concept, the higher their quality. The original Q‐DIO scale was validated in several studies (Linch et al., 2015; Müller‐Staub et al., 2009, 2010). Internal consistency by Cronbach's alpha ranged from 0.83–0.99 for each sub‐concept, test–retest reliability by kappa was 0.95 and interrater reliability by Fleiss' kappa was 0.94.

Standardized data collection charts were used to determine potential confounders. Hereby, data on nurses' gender, their highest education grade, years of practical experience and previous Advanced Nursing Process education were collected. From each ward, data on bed occupancy rate, patients' LOS, nurse‐to‐patient ratio (calculation based on full‐time equivalents), skill‐ and grade‐mix, staff turnover and characteristics of the nursing care delivery system (e. g. autonomy and authority about nursing decisions, communication) were collected.

#### 3.2.2 Procedure

Nurses completed the PND and the knowledge test on their wards and ward managers/Advanced Practice Nurses gave instructions on applying the test (e. g. answering all questions, time limit, submitting). After explaining, first the PND was silently completed by hand, requiring five minutes. The PND's were coded using a personal four‐digit number (e.g. last phone digits) to assure anonymity and comparison with their knowledge tests. Second, the knowledge test was silently taken by handwriting. Its completion allowed a maximum of 30 min. The participants were asked to fill in the same four‐digit number as before. The response sheets were collected in a large envelope and closed by the last participant. The knowledge tests were assessed by the first author according to standardized responses and standard scores as outlined by the author of the test.

In the nursing records, each of the 35 Q‐DIO R items was evaluated by the first author. To ensure a consistent approach, the instrument developer also evaluated several records and comparisons were made. If evaluations differed, consensus was sought and correct instrument application was assured by writing memos.

Nurses' characteristics were collected with the PND, patient characteristics from the nursing records and the principal investigator charted organizational data from all six wards.

### 3.3 Data analysis

All data were analysed by IBM SPSS Statistics 24 (SPSS Inc.). Descriptive statistics were performed for frequencies and distribution of sample characteristics and outcome measures (nurses' knowledge, attitude, quality of the Advanced Nursing Process). Inductive statistics (Kruskal–Wallis tests) were used to compare the outcome measures between the three departments (medical, surgical and acute geriatric). Repeated post hoc tests between groups were conducted including Bonferroni correction (Clauß, Finze, & Partzsch, 2011). Comparisons based on single items and on total scores of the knowledge tests and self‐assessments of nurses' attitude (PND). The Q‐DIO R scores were summed up for each sub‐concept (Müller‐Staub et al., 2010). Pearson's product‐moment correlations were used to determine the relations between metrical data (knowledge scores, Q‐DIO R scores, patient characteristics, e.g. age and LOS and organizational factors). The relations between ordinal data (nurses' attitude, educational degree and practical experience) or between ordinal and metric data were analysed by Spearman correlations. Chi‐squared tests were used to investigate associations between patients' gender and for previous Advanced Nursing Process education with other variables (LOS, knowledge, attitude and Q‐DIO R sub‐concepts). Linear regression analyses were performed to determine how well a dependent variable (e. g. quality of nursing outcomes) could be explained by independent variables (e. g. accuracy of NDs, effectiveness of nursing interventions) if the statistical prerequisites were attained. All tests were applied one‐sided (significance level 0.05) (Bortz & Schuster, 2016).

### 3.4 Ethical considerations

Ethics approval was obtained from the responsible cantonal committee (Nr. PB_2016_00990). The study was executed in conformity with the Declaration of Helsinki (WMA, 2013) and with the study country's laws and regulations. The nurses from the six hospital wards were asked for voluntary participation and gave informed consent.

### 3.5 Validity and reliability/Rigour

As described in the instrument section, the PND and Q‐DIO were psychometrically tested and showed good results. In a pilot application, the knowledge test was evaluated by experts and modified before application. Additionally, the research team was trained to assure standardized data collection and the STROBE Statement (Appendix S1) was used for reporting (Elm et al., 2007).

## 4 RESULTS

Ninety‐nine RNs were included, 92 returned the PND (response rate: 92.9%). Most of the nurses were female, had a tertiary (academic) degree and one third had more than 21 years of practical experience (Table 1). Patient characteristics in nursing records and departments are shown in Table 2. Two thirds of patients were female, acute geriatric patients were on average older, and their average LOS was nearly doubled compared with medical and surgical patients.

**Table 1:** Main characteristics of nurses (*N* = 92)

Gender		Advanced nursing process education		Grade		Practical experience in years	
Female	82 (89.1%)	Yes	42 (45.7%)	RN Diploma	65 (70.7%)	0–5	20 (21.7%)
Male	10 (10.9%)	No	50 (54.3%)	Diploma and Advanced Studies (+15–30 ECTS)	15 (16.2%)	6–10	14 (15.2%)
				BSc	3 (3.3%)	11–15	15 (16.3%)
				MSc	1 (1.1%)	16–20	11 (12.0%)
				Management training	8 (8.7%)	>21	32 (34.8%)

Note

ECTS, European Credit Transfer System, 1 ECTS, 25 hr participants' learning effort.

**Table 2:** Characteristics of included patients

Patients *N* = 90	Gender		Age in years		Length of stay	
	Female	Male	Mean (*SD*)	Min; max	Mean (*SD*)	Min; max
Acute geriatric wards (*N* = 30)	20 (66.7%)	10 (33.3%)	82.6 (8.4)	66; 98	17.8 (8.2)	5; 44
Medical wards (*N* = 30)	22 (73.3%)	8 (26.7%)	73.1 (17.6)	27; 93	9.9 (3.8)	4; 22
Surgical wards (*N* = 30)	19 (63.3%)	11 (36.7%)	69.0 (16.9)	36; 94	10.0 (4.6)	5; 28

*Research question 1: How is nurses' knowledge and attitude towards the* Advanced Nursing Process*?*

Thirty‐three of 34 nurses completed the knowledge test. The total score of the six knowledge questions ranged from 2.0–30.5 scores, corresponding to a mean of 16.2 (*SD* 7.0). The best response was achieved on the definition and function of the PES format. The self‐assessment of nurses' understanding indicated low knowledge levels [4.0 scores (*SD* 1.9)] and supported the total scores [*r* = 0.365 (*p* = .066)]. The RNs of both medical wards showed 30% higher scores in the knowledge test than the acute geriatric nurses [18.4 (*SD* 3.7) vs. 12.5 (*SD* 7.4); *p* = .020]. The surgical nurses reached 17.9 (*SD* 7.4) scores.

Nurses' attitude showed an average of 5.4 (*SD* 0.8) scores on the PND scale (from 0–7). The Advanced Nursing Process was judged being very meaningful [6.1 (*SD* 1.1)], very important [6.1 (*SD* 1.0)], very positive and rewarding [both 5.8 (*SD* 1.1)]. However, RNs rated it being uncomfortable [3.9 (*SD* 1.4)], difficult [4.2 (*SD* 1.4)] and trivial [4.5 (*SD* 1.2)]. No statistically significant differences were found between the three departments [acute geriatric wards: 5.4 (*SD* 0.8), medical wards: 5.5 (*SD* 0.7) and surgical wards: 5.3 (*SD* 0.9), (*p* = .715)]. Nevertheless, medical nurses with higher educational levels showed more positive attitudes [*r* = 0.343 (*p* = .086)].

*Research question 2: What is the quality of the* Advanced Nursing Process *as measured by (a) the accuracy of NDs, (b) effectiveness of nursing interventions, (c) the quality of nursing outcomes and d) frequency and variety of NDs?*

The quality of nursing assessments (Q‐DIO R sub‐concept one) ranged from 0.3–1.8 [1.2 (*SD* 0.3)] on a scale from 0–2. The accuracy of NDs as evaluated by the PES format and its accordance with nursing assessment and nursing notes' data and was 2.3 (*SD* 0.6) (range: 0.4–3.5) on a scale from 0–4. The effectiveness of nursing interventions (e.g. intervention effects, achieving expected outcomes and clarity of description) ranged from 0.6–3.3 [1.9 (*SD* 0.6)]. Expected patient outcomes were achieved and demonstrated intervention effectiveness with 1.9 (*SD* 0.6) (range: 0.5–3.6). The highest rated quality of NDs, interventions and nursing‐sensitive patient outcomes were attained on both medical wards (Table 3). There were significant differences in the quality of nursing interventions between the medical and surgical wards (*p* = .014) as well as in the quality of nursing outcomes between the medical and acute geriatric wards (*p* = .002).

**Table 3:** Quality of nursing diagnoses, interventions and outcomes within the three departments

*N* = 90	Nursing assessment (0–2)	Nursing diagnosis (0–4)	Nursing interventions (0–4)	Nursing outcomes (0–4)
Acute geriatric wards (*N* = 30)	1.27 (*SD* 0.20)	2.31 (*SD* 0.52)	1.84 (*SD* 0.58)	1.67 (*SD* 0.53)
Medical wards (*N* = 30)	1.26 (*SD* 0.27)	2.42 (*SD* 0.55)	2.14 (*SD* 0.59)^c^	2.10 (*SD* 0.63)^b^
Surgical wards (*N* = 30)	1.14 (*SD* 0.29)	2.24 (*SD* 0.81)	1.78 (*SD* 0.54)	1.88 (*SD* 0.69)

The results of b and c were tested by Kruskal‐Wallis test

^b^ Level of significance 0.01.

^c^ Level of significance 0.05.

**Figure 1:**
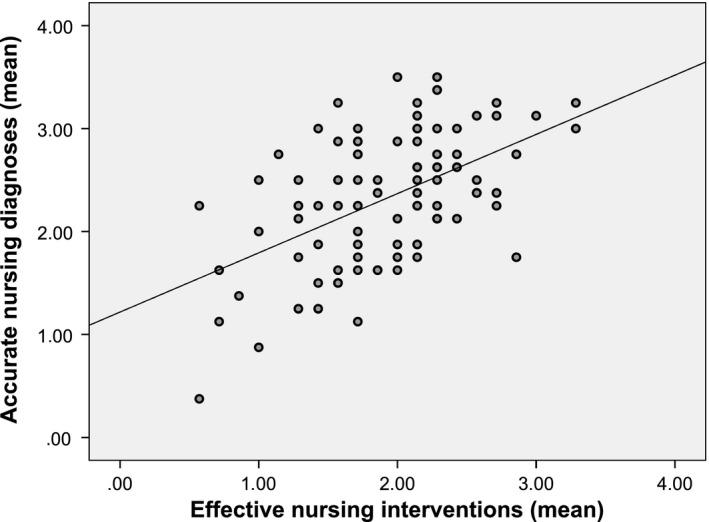
Significant Pearson's correlation between accurate nursing diagnoses and effective nursing interventions (*N* = 90) [*r* = 0.528 (*p* < .0001)]; linear regression model [*R^2^* = 0.279, *F* = 34.029, *p* < .001]

**Figure 2:**
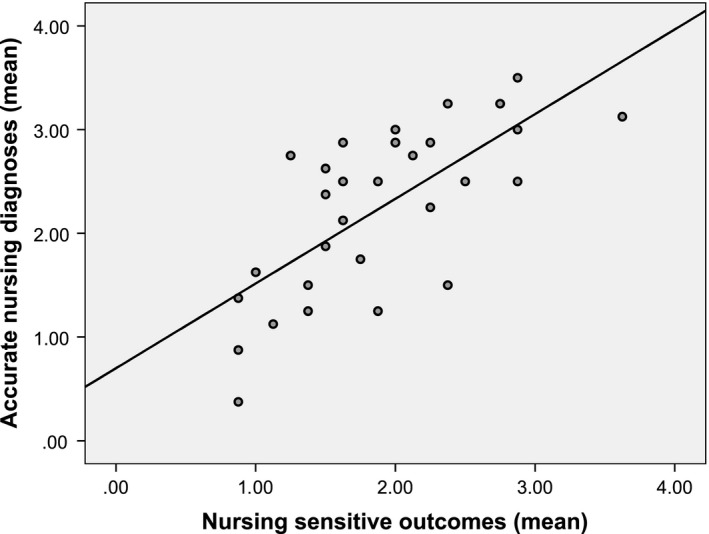
Significant Pearson's correlation between accurate nursing diagnoses and nursing‐sensitive outcomes at two surgical wards (*N* = 30) [*r* = 0.622 (*p* < .001)]; linear regression model [*R* ^2^ = 0.386, *F* = 55.426, *p* < .001]

The internal coherence and thus the correlation between NDs, interventions and nursing‐sensitive patient outcomes was strong and highly significant. The higher the accuracy of NDs the more effective the nursing interventions [*r* = 0.528 (*p* < .0001)]. The effectiveness of nursing interventions can be declared with 28% by the accuracy of NDs (*p* < .001) (Figure 1). Accurate NDs were also strongly linked to better nursing‐sensitive patient outcomes [*r* = 0.622 (*p* < .001)]. This is shown in Figure 2 with reference to two surgical wards [*r* = 0.701 (*p* < .0001)]. Across all wards, the quality of outcomes can be declared with 39% by the accuracy of NDs (*p* < .001). Besides, the more effective the nursing interventions, the better were the nursing‐sensitive patient outcomes [*r* = 0.576 (*p* < .001)]. The accuracy of NDs (*ß* = 0.440, *p* < .001) jointly with the effectiveness of nursing interventions (*ß* = 0.343, *p* < .001) explain the quality of nursing‐sensitive patient outcomes to 46% (*corr. R* ^2^ = 0.459, *F*(2) = 38,778, *p* < .001). The standardized regression coefficients (*ß*) show that the variance of the quality of nursing outcomes can be explained by the accuracy of NDs more than by the effectiveness of nursing interventions. In Table 4, the first two examples show accurate NDs with correctly linked interventions and outcomes (=strong correlation). An inaccurate ND, expected outcomes that did not build a bridge between NDs and interventions and nursing interventions which did not focus on taking over, assistance or guidance for bathing and dressing—as an example of lacking coherence—is described in the lowest line in Table 4.

**Table 4:** Examples of accurate/inaccurate nursing diagnoses linked with interventions and nursing‐sensitive outcomes

	Nursing diagnosis	Expected outcomes	Nursing interventions	Nursing‐sensitive patient outcomes
**+**	P: Ineffective breathing pattern E: Tracheobronchial infection, chronic pulmonary disease S: Dyspnoea, tachypnea, cough, reduced activity tolerance	Breathing normally and effectively	Nurses ‐ Identify the breathing pattern ‐ Administer oxygen according medical orders ‐ Observe the presence of secretion ‐ Support the patient to adhere rest phases ‐ Support the patient in daily activities	Patient still has exertional dyspnoea; however, he can walk 20 m without a break; when resting, he reports no complaints
	P: Acute pain E: Inflammatory process, metastatic prostate cancer S: General backache and hip pain radiating into the legs	‐ Normalization of motion sequences and mobilization ‐ Expressing pain reduction	Nurses ‐ Determine pain (pain scale) once per shift ‐ Evaluate the pain medication during visits ‐ Administer additional pain medication (reserve orders) as needed	Pain medication was readjusted. By now, the patient expresses noticeably fewer pain. Pain medication from the reserve was requested by the patient periodically; it seems, he can handle it
**‐**	P: Bathing and dressing self‐care deficit (E and S were missing)	‐ Patient provides himself well at home, house‐keeping is manageable ‐ Patient can accept the help that she currently sees as unnecessary	‐ The physician talks with the family caregiver, so that the patient receives their support or that they organize home care ‐ Home help would be desirable, because the home/household is in a messy stage	This morning, the patient took a shower independently, she was doing well

In the care plans, 34 different NDs were documented. The variety of NDs on the medical wards (*N* = 27) was noticeably larger than on the surgical wards (*N* = 17). On average, patients had 2.6 NDs (range: 1–9). The average number of NDs was significantly higher in acute geriatric patients [3.1 (*SD* 1.8)] compared with patients on the medical [2.3 (*SD* 1.7)] or surgical wards [2.3 (*SD* 1.3), *p* = .053)]. The most frequent NDs focused on functional impairments and related risks: risk for falls (*N* = 37), self‐care deficit bathing and dressing (*N* = 32) and impaired physical mobility (*N* = 19). Acute pain—as an unpleasant sensory and emotional experience—was the most frequently (*N* = 21) documented ND.

*Research question 3: Do correlations exist between nurses' knowledge and attitude with the quality of the* Advanced Nursing Process*?*

Nurses' knowledge was statistically significantly related with the quality of the Advanced Nursing Process. On the surgical wards, the quality of nursing assessments was better in nurses with higher knowledge [*r* = 0.395 (*p* = .031)]. On the medical wards, higher nurses' knowledge related to higher accuracy of NDs [*r* = 0.502 (*p *= .005)] and to better nursing outcomes [*r* = 0.369 (*p* = .045)]. There was no relation found between nurses' knowledge and a previous Advanced Nursing Process education [*χ^2^* = 0.008 (*p* = .930)].

The quality of nursing assessments correlated with nurses' attitude. Nurses with more positive attitudes completed more complete and more exact nursing assessments [*r *= 0.287 (*p* = .006)]. However, nurses' attitude was not associated with the extent of their practical experience years [*r* = −0.010 (*p* = .924)], nor with their educational degrees [*r* = 0.136 (*p* = .196)]. However, a connection was found between more positive nurses' attitudes and higher knowledge levels [*r* = 0.325 (*p* = .065)].

*Research question 4: Do correlations exist between patient characteristics and the quality of the* Advanced Nursing Process*?*

Significant correlations were found between patients' age, LOS and the quality of the Advanced Nursing Process.

If elderly patients stayed longer in the hospital, the quality of the nursing assessment and the number of NDs were higher. Old age was strongly related to better nursing assessments on surgical wards [*r* = 0.649 (*p* = .009)] and on medical wards [*r* = 0.625 (*p* = .013)]. A similarly strong relation between high‐quality nursing assessments and prolonged LOS was found on acute geriatric [*r* = 0.559 (*p* = .030)] and surgical wards [*r* = 0.544 (*p* = .036)]. The number of NDs was significantly higher, when patients were older [*r* = 0.325 (*p* = .002)] and when they remained longer in the hospital [*r* = 0.556 (*p* < .0001)]. For example, an average of 2.8 (*SD* 1.5) NDs was found in patients of age 80–89 years and 3.5 (*SD* 2.3) NDs in patients that were older than 90 years. Patients with LOS ≤ 8 had 1.9 ND (*SD* 0.9), LOS ≤ 19 = 2.5 NDs (*SD* 1.5) and patients with LOS ≥ 20 = 4.2 NDs (*SD* 2.0).

Patient age and LOS were also significantly related with nursing‐sensitive patient outcomes. The younger the surgical patients, the shorter their' LOS and the better were their nursing‐sensitive patient outcomes [age: *r* = −0.433 (*p* = .017); LOS: *r* = −0.261 (*p* = .013)]. Gender was not related to the quality of the Advanced Nursing Process [e.g. to the quality of ND: *χ^2^* = 0.696 (*p* = .706)].

Further, we found out that the skill‐ and grade‐mix was statistically significantly related with the quality of the Advanced Nursing Process. Higher proportions of RNs in nursing teams showed more complete/specific nursing assessments [*r* = 0.264 (*p* = .012)] and better nursing‐sensitive patient outcomes [*r* = 0.354 (*p* = .001)]. None of the other organizational factors (Table 5) were statistically significantly related with the ND' quality.

**Table 5:** Correlations between patient characteristics, organizational factors and nursing diagnosis quality (*N* = 90)

Pearson's correlation	*r*	*p*
Patients' age	−.171	.106
Length of stay	−.038	.724
Bed occupancy rate	−.085	.428
Nurse‐to‐patient ratio	.113	.290
Skill‐ and grade‐mix	.135	.205
Staff turnover	−.038	.720

## 5 DISCUSSION

### 5.1 Synopsis of main results

The overall analyses of knowledge and attitude tests, as well as of nursing records demonstrated rather low levels of nurses' knowledge of, a positive attitude towards and an average quality of the Advanced Nursing Process. The quality of the nursing assessment and ND was slightly above the Q‐DIO score average, while the quality of nursing interventions and outcomes were slightly below the mean. A strong relationship was found between the quality of ND, interventions and outcomes. The more accurate the ND, the more effective the interventions and the better the nursing‐sensitive patient outcomes. This demonstrates that accurate NDs are key and the starting point for the next phases of the Advanced Nursing Process (e.g. choosing effective interventions to attain favourable patient outcomes). The nurses of the medical wards had more knowledge, a slightly more positive attitude and the quality of ND, interventions and outcomes as well as the variety of NDs were the highest compared with the other wards. The higher the proportion of RNs within a team was, the better were nursing‐sensitive patient outcomes.

### 5.2 Nurses' knowledge and attitude

When looking specifically at these variables, nurses' knowledge towards the Advanced Nursing Process was limited. A partial explanation may be that almost half of the RNs' basic education dated back more than 15 years. RNs of Greek primary healthcare settings with similar years of experience (Patiraki et al., 2017) and Ethiopian nurses (Kebede et al., 2017) also showed lack of knowledge. In contrast, nursing Bachelor students (more than half of them had a previous nursing diploma) showed good knowledge about using NDs (El‐Rahman, Al Kalaldeh, & Malak, 2017).

The RNs showed positive attitudes towards NDs (PND $\bar{x}$ = 5.4). Previous studies indicate that the Swiss nurses ($\bar{x}$ = 5.4) (Leoni‐Scheiber, 2013) were amongst the leaders compared with other European nurses. Austrian nurses ($\bar{x}$ = 5.4) (Leoni‐Scheiber et al., 2016), Italian nurses ($\bar{x}$ = 5.1) (D'Agostino et al., 2016), Spanish nurses ($\bar{x}$ = 4.9) (Romero‐Sanchez et al., 2013) and German nurses ($\bar{x}$ = 4.8) demonstrated lower attitude scores (Leoni‐Scheiber et al., 2016). The positive attitude of the Swiss nurses in this hospital might stem from the initial implementation of NDs thirteen years ago as well as from the ongoing management and APN support. Nevertheless, nursing diagnostics was seen as difficult. Clinical decision‐making including diagnostic reasoning is not an easy task. Interviews with members of the American Academy of Nurse Practitioners (*N* = 731) showed that 25% had difficulties in documenting nursing care, whereas lack of knowledge about the Advanced Nursing Process and unfamiliarity with nursing classifications were reasons for these results (Conrad et al., 2012).

### 5.3 Quality of ND, interventions and outcomes

The mean Q‐DIO scores of the quality of NDs ($\bar{x}$ = 2.3), interventions ($\bar{x}$ = 1.9) and outcomes ($\bar{x}$ = 1.9) ranked in the lower midfield compared with previous studies. Before an initial implementation in a Swiss hospital in 2003, NDs were also less accurate ($\bar{x}$ = 0.92), interventions less effective ($\bar{x}$ = 1.27) and nursing outcomes not satisfactory ($\bar{x}$ = 0.95). After this implementation project, the Q‐DIO scores increased significantly (NDs $\bar{x}$ = 2.91, interventions $\bar{x}$ = 2.51 and outcomes $\bar{x}$ = 1.78) (Müller‐Staub et al., 2009). After implementing NANDA‐I NDs and NIC interventions in two Brazilian hospitals, the nurses stated more accurate NDs ($\bar{x}$ = 2.7), performed more effective interventions ($\bar{x}$ = 3.0) and reached better nursing‐sensitive patient outcomes ($\bar{x}$ = 1.7) (Rabelo‐Silva et al., 2016).

Also, Kebede et al. (2017) support our results: A higher Advanced Nursing Process quality was found on medical wards compared with surgical ones. In contrast, in a Nigerian university hospital the Q‐DIO total scores were significantly higher in surgical than in medical wards (Adubi, Olaogun, & Adejumo, 2017).

We identified a strong and highly significant correlation between the quality of ND, effectiveness of interventions and better nursing‐sensitive patient outcomes. Other studies support these results. In a Brazilian hospital, where NANDA‐I and NIC were used, the accuracy of ND, the effectiveness of nursing interventions and the outcomes were significantly better compared with another hospital where the International Classification for Nursing Practice (ICNP) was applied (Rabelo‐Silva et al., 2016). The ICNP does not contain a comprehensive description of concepts (PES format) and theory‐based linkages between ND and nursing interventions and outcomes are missing (Müller‐Staub et al., 2017). The quality of nursing outcomes was also influenced by the number of NDs: fewer NDs were statistically significantly associated with better nursing‐sensitive outcomes (Scherb et al., 2013) while patients with a higher number of NDs tended to be in worse general conditions (Castellan, Sluga, Spina, & Sanson, 2016; D'Agostino et al., 2017).

### 5.4 Frequency and variety of NDs

The number of NDs per patient, the variety of and the most frequent NDs depend on the patient (group), setting/discipline and on organizational factors (e.g. software application). The average number of NDs per patient in our study was 2.6. All previous studies showed more than 3.5 [from 3.8 (Aguilar & Pancorbo‐Hidalgo, 2011) to 9.6 (Araúja, Carvalho, & Chianca, 2014)]. The variety of NDs depended on the number of NDs that were contained in the EHR. In the EHR that was used in the study site, only 43 NDs—out of totally 245 NANDA‐I NDs—were available. From these, 31 were used, twelve were not used and only two additional ones were stated. In a study with elderly patients undergoing surgery, 14 of 74 possible NDs were used (Tuncbilek & Celik, 2016). The most frequent NDs in this study were congruent with previous studies in differing rankings and combinations: risk for falls, risk for infection, acute pain, fear, impaired physical mobility, self‐care deficit bathing and dressing (D'Agostino et al., 2017; Paans & Müller‐Staub, 2015; Tuncbilek & Celik, 2016). A crucial factor for the variety of NDs may be which and how many NDs are provided by the EHR.

### 5.5 Correlations between nurses' knowledge, attitude and the quality of the Advanced Nursing Process

Nurses' knowledge was associated with the quality of the Advanced Nursing Process. After training, nurses identified more accurate NDs (Pobocik, 2015; Predebon et al., 2012). Other factors, such as a predefined record structure with the PES format, were significantly associated with more accurate NDs (Paans, Sermeus, Nieweg, Krijnen, & Schans, 2012); and three studies reported that participants of nursing process education had a more positive attitude towards NDs (Collins, 2013; D'Agostino et al., 2016; Romero‐Sanchez et al., 2013).

Higher knowledge on and positive attitudes towards the Advanced Nursing Process were associated with better nursing process quality (Kebede et al., 2017). In our study, solely the nursing assessment quality revealed a positive, significant correlation with nurses' attitude.

Our results are supported by three studies were positive correlations between nurses' knowledge and attitude were reported [El‐Rahman et al. (2017) [*r* = 0.445 (*p* < .001)], Ogunfowokan, Oluwatosin, Olajubu, Alao, and Faremi (2013) (*p* = .04) and Oliva, Lopes, Volpato, and Hayashi (2005). In contrast, Patiraki et al. (2017) did not find a connection between nurses' attitude and their skills regarding ND formulation.

### 5.6 Correlations between patient characteristics and the quality of the Advanced Nursing Process

Higher patient age and longer LOS were associated with a higher number of NDs per patient and less positive nursing‐sensitive outcomes. In previous studies, nurses stated also more NDs in elderly patients [6.1 (Heering, 2011) and 9.6 (Araúja et al., 2014)]. Two large‐scale studies revealed statistically positive correlations between numbers of NDs and LOS (D'Agostino et al., 2017; Welton & Halloran, 2005). Our correlations to nursing outcomes match with US‐American results (Scherb et al., 2013): the longer the LOS of patients aged from 60–89, the less improvements in several nursing‐sensitive outcomes (e.g. knowledge) were found. These facts are not surprising, as old(er) patients show multimorbidities/progressive diseases/increased complications and associated impairments as well as longer LOS.

A higher proportion of RNs per ward was associated with more comprehensive nursing assessments and better nursing‐sensitive patient outcomes. This finding is in line with previous studies in US‐American and European hospitals (Aiken et al., 2011; Kutney‐Lee, Lake, & Aiken, 2009).

### 5.7 Limitations

The results of this study are transferable to a limited extent because of the small sample size (knowledge test) and the sampling procedure (convenience sampling for knowledge tests). The reasons for sampling were the size of the hospital and the availability of organizational resources for this study. Due to missing statistical pre‐requirements (e. g. normal distribution of the dependent variable, multicollinearity), multilevel regression analyses could be applied not for all variables. Despite this, the sample size of nursing records, the multiple foci of measurements and the comparability of departments allow reliable conclusions.

## 6 CONCLUSIONS

The aim of this study was to detect possible relationships between nurses' knowledge, attitude, patient characteristics and the Advanced Nursing Process quality. Nurses' knowledge and positive attitudes are key for a good Advanced Nursing Process quality. Better nursing assessments, more accurate NDs and better nursing‐sensitive patient outcomes were associated with good knowledge. Nurses need good knowledge to state NDs in the correct PES format, to derive appropriate nursing interventions from the NDs' aetiological or risk factors and to link them with relevant and fitting patient outcomes. The measured quality of nursing assessment, diagnoses, interventions and outcomes was on average, compared with other studies. We conclude that nurses should be supported to develop their knowledge and clinical decision‐making competencies.

Relationships between patients' higher age, longer LOS, higher number of NDs and less favourable nursing‐sensitive patient outcomes provide clues on the severity of patients' health conditions. In hospitals, there is a tendency for increasing numbers of elderly patients in complex situations. Furthermore, the quality of nursing assessments and patient outcomes was better in wards with higher proportions of RNs. These factors should be considered, as it is known that good nurse‐to‐patient ratios are leading to better nursing care.

To our knowledge, this is the first correlational study reporting strong relationships between the quality of NDs, intervention effectiveness and better nursing‐sensitive patient outcomes. Further research using diverse research methods, larger samples in different settings and additional statistical evaluations such as multilevel regression analyses are warranted to address relations and effects when applying the Advanced Nursing Process.

## CONFLICT OF INTEREST

No conflict of interest has been declared by the author(s).

## AUTHOR CONTRIBUTIONS

CLS, HM, MMS: Have made substantial contributions to conception and design, or acquisition of data, or analysis and interpretation of data. CLS, HM, MMS: Been involved in drafting the manuscript or revising it critically for important intellectual content. HM, MMS: Given final approval of the version to be published. Each author should have participated sufficiently in the work to take public responsibility for appropriate portions of the content. CLS, HM, MMS: Agreed to be accountable for all aspects of the work in ensuring that questions related to the accuracy or integrity of any part of the work are appropriately investigated and resolved.
